# Evaluating a Custom Chatbot in Undergraduate Medical Education: Randomised Crossover Mixed-Methods Evaluation of Performance, Utility, and Perceptions

**DOI:** 10.3390/bs15091284

**Published:** 2025-09-19

**Authors:** Isaac Sung Him Ng, Anthony Siu, Claire Soo Jeong Han, Oscar Sing Him Ho, Johnathan Sun, Anatoliy Markiv, Stuart Knight, Mandeep Gill Sagoo

**Affiliations:** Faculty of Life Sciences and Medicine, King’s College London, London WC2R 2LS, UK

**Keywords:** artificial intelligence, medical education, Large Language Models, chatbots, learning perceptions, student engagement, cognitive load, educational technology

## Abstract

Background: While LLM chatbots are gaining popularity in medical education, their pedagogical impact remains under-evaluated. This study examined the effects of a domain-specific chatbot on performance, perception, and cognitive engagement among medical students. Methods: Twenty first-year medical students completed two academic tasks using either a custom-built educational chatbot (Lenny AI by qVault) or conventional study methods in a randomised, crossover design. Performance was assessed through Single Best Answer (SBA) questions, while post-task surveys (Likert scales) and focus groups were employed to explore user perceptions. Statistical tests compared performance and perception metrics; qualitative data underwent thematic analysis with independent coding (κ = 0.403–0.633). Results: Participants rated the chatbot significantly higher than conventional resources for ease of use, satisfaction, engagement, perceived quality, and clarity (*p* < 0.05). Lenny AI use was positively correlated with perceived efficiency and confidence, but showed no significant performance gains. Thematic analysis revealed accelerated factual retrieval but limited support for higher-level cognitive reasoning. Students expressed high functional trust but raised concerns about transparency. Conclusions: The custom chatbot improved usability; effects on deeper learning were not detected within the tasks studied. Future designs should support adaptive scaffolding, transparent sourcing, and critical engagement to improve educational value.

## 1. Introduction

Recent advancements in artificial intelligence (AI), particularly following the public deployment of Large Language Models (LLMs) such as ChatGPT, have influenced diverse sectors, including healthcare and education. Within educational contexts, these AI-driven technologies offer new opportunities to support learning and enhance knowledge acquisition. While clinical applications of AI, ranging from diagnostic algorithms to decision-support systems, are well-documented (Wartman & Combs, 2017; Banerjee et al., 2021), there is comparatively less empirical work investigating how LLM-powered tools affect the ways medical students acquire and apply knowledge.

While AI applications in healthcare delivery are well documented, our focus here is on AI in medical education, where the evidence base is more limited but rapidly emerging. Although interest in LLM chatbots in medical education has accelerated, much of the literature has focused primarily on tool validation and user experience, while conflating interface appeal with pedagogical effectiveness and offering less analysis grounded in theory or learning outcomes. Moreover, current research indicates that while medical students increasingly acknowledge AI’s significance, they often feel inadequately prepared to engage with it in clinical or educational contexts (Sit et al., 2020). A recent scoping review highlighted a persistent lack of empirical work evaluating the impact of AI tools on learning experience, knowledge retention, and higher-order cognitive skills (Gordon et al., 2024), thereby limiting insight into their educational value and theoretical coherence.

While students often express optimism about AI’s potential in medical education, multiple studies suggest that their understanding of its practical applications and limitations remains superficial (Amiri et al., 2024; Jebreen et al., 2024). This may, in part, reflect the lack of structured AI education within medical curricula, which has been shown to negatively affect students’ conceptual grasp and critical appraisal of AI tools (Pucchio et al., 2022; Buabbas et al., 2023). As a result, it remains difficult to assess whether AI-assisted learning offers substantive educational advantages over conventional methods. Although some studies have reported improvements in engagement and accessibility in resource-constrained settings, the extent to which AI fosters critical thinking and deeper understanding remains unclear (Jackson et al., 2024; Salih, 2024; Civaner et al., 2022; Jha et al., 2022; Luong et al., 2025).

Notably, there are recent evaluations that focus on better understanding how medical students interact with AI in learning contexts. Kochis et al. (2024) reported that students adopted chatbots primarily as supplementary tools, with mixed perceptions of accuracy and reliability. Arun et al. (2024) showed that domain-specific tailoring can outperform generic LLMs in anatomy tasks, although the gains were modest and context-dependent. Araujo and Cruz-Correia (2024) highlighted the challenges of integrating ChatGPT into medical curricula, with issues of curricular alignment and student trust emerging as central themes. Lucas et al. (2024) synthesised this emerging field in a systematic review, emphasising that while usability and efficiency are often reported, evidence for sustained deep learning remains sparse. Taken together, these early evaluations suggest that AI chatbots improve usability and engagement but show variable impact on depth and performance.

In the hope of designing chatbots that can consistently have a positive impact on the depth of learning and performance scores, we considered educational theories and principles that could be useful to integrate. Guided by cognitive frameworks, these tools can provide instant feedback, clarify complex concepts, and scaffold clinical problem-solving. For instance, Sweller’s Cognitive Load Theory (CLT) emphasises the importance of minimising extraneous processing demands to promote deep learning (Sweller, 2011). Incorporating this into chatbot design may help students engage with complex content more effectively without becoming overloaded (Gualda-Gea et al., 2025), which could be reflected in students’ perceptions when using such tools. Another conceptual framework that we referenced is the Dual Process Theory, which differentiates between intuitive (System 1) and analytical (System 2) cognition, offering a lens to assess how chatbots may facilitate rapid recall while potentially limiting reflective reasoning (Evans & Stanovich, 2013). This aligns with our purpose to better understand chatbot-assisted learning, specifically whether it fosters surface-level recall only or supports more deliberate conceptual integration. This could manifest as a discordance between students’ perceived usefulness of LLM chatbots and their actual performance when faced with questions that test high-order thinking. In addition, the Technology Acceptance Model (TAM) introduces a behavioural perspective, highlighting the role of perceived usefulness and ease of use in shaping students’ adoption of educational technologies. Lastly, Epistemic Trust Theory provides a foundation for analysing student perceptions of the transparency, credibility, and reliability of AI-generated information (McCraw, 2015). This is especially relevant when exploring student attitudes toward AI in education and whether they perceive the chatbot as reliable and aligned with curricular expectations.

Incorporating these frameworks and in response to the limitations of generic tools, this study aims to evaluate “Lenny AI”, a custom GPT-4o-based educational chatbot aligned to the UK undergraduate anatomy curriculum. Lenny is built by the authors of the study, and differs from generic models through prompt guardrails, conservative sampling, structured outputs and mnemonic summaries, all intended to reduce extraneous load and improve curricular fit. Our study will evaluate Lenny AI quantitatively and qualitatively based on the following research hypotheses (1–4) and questions (5–8):

Hypothesis (Directional):

1. Consistent with the Technology Acceptance Model (TAM), participants will report significantly higher scores in the measured perception parameters (such as ease of use, satisfaction, engagement and perceived usefulness) when using the LLM chatbot compared to conventional study tools.
2. Informed by the Cognitive Load Theory (CLT), the use of the LLM chatbot will result in higher SBA performance scores compared to conventional tools, reflecting LLMs’ abilities to reduce extraneous cognitive load.
3. According to Dual-Process Theory, Chatbot use will primarily enhance performance in questions targeting rapid factual recall and boost confidence in applying information (System 1 processing), but will not significantly enhance ratings of depth of content or critical thinking (System 2 processing).
4. Based on the Epistemic Trust theory, students will demonstrate high functional trust in chatbot outputs (accuracy, reliability), while also expressing reservations about transparency and alignment with curricular expectations.

Research Questions (Exploratory):

5. How do medical students perceive the usefulness and usability of LLM chatbots compared to conventional study tools?
6. What are students’ experiences with LLM chatbots in supporting their learning, engagement, and information retention?
7. How do students perceive the limitations or challenges of using LLM chatbots for medical studies?
8. What changes, if any, do students report in their attitudes toward AI in medical education after using the chatbot?
9. To what extent do students feel the chatbot aligns with their curriculum and supports deeper learning and critical thinking?

Further implementation details are described in the Materials and Methods section.

## 2. Materials and Methods

### 2.1. Study Design

The study design was informed by two theoretical frameworks. First, the Technology Acceptance Model (TAM) guided our choice of perception measures. TAM emphasises perceived ease of use and perceived usefulness as central to adoption (Davis, 1989). We therefore included survey items on usability, satisfaction, engagement, confidence, and perceived quality, which correspond to these constructs. Second, Dual-Process Theory shaped our attention to different modes of reasoning. Single Best Answer (SBA) tasks provided a structured test of factual retrieval and applied reasoning under time limits, while post-task surveys and focus group discussions probed students’ reflections on transparency, trust, and deeper cognitive engagement. This combination allowed us to capture both surface-level fluency and opportunities for analytic processing. Together, these frameworks structured the measures and informed the interpretation of results.

This study employed a randomised controlled crossover design to evaluate the educational impact of an LLM chatbot (Lenny AI) compared to conventional study materials in preclinical, undergraduate medical education. Each participant completed two academic tasks, experiencing both the AI-supported and conventional learning conditions in alternating order. The study combined quantitative scores and survey measures with qualitative data from post-intervention focus group discussions, allowing for a mixed-methods analysis of both perceived and objective learning outcomes. The main research questions that we aim to answer are listed in the Introduction section.

### 2.2. Participants and Setting

A total of 20 first-year medical students from GKT School of Medical Education, King’s College London (KCL), participated in the study. Eligible participants were enrolled in the standard five-year Medicine MBBS Programme and had completed a minimum of three months of preclinical instruction. Students on the Postgraduate Entry Programme or the Extended Medical Degree Programme were excluded. Participants were recruited via posters and offered a small token of appreciation for their time, in accordance with institutional policy and ethics approval. All participants completed the full study protocol.

The study was conducted face-to-face in a classroom setting using facilities provided by KCL in 2024. To ensure standardisation, all participants accessed materials via university-provided computers or pre-prepared physical handouts.

### 2.3. Study Materials

#### 2.3.1. Conventional Study Materials

For the control condition, participants used conventional learning resources, including anatomical diagrams, concise explanatory texts, and summary tables, reflecting the typical content format encountered in undergraduate anatomy teaching at KCL. Materials were derived from standardised textbook excerpts mapped to the relevant task topics:

1. Clinical Anatomy: Applied Anatomy for Students and Junior Doctors (Ellis & Mahadevan, 2019).
2. Clinically Oriented Anatomy (Moore et al., 2017).

These materials were printed and distributed as handouts during the study session. In addition, students were permitted to use university computers to consult non-AI digital resources, such as Google search or medical websites, consistent with typical self-directed study. However, all AI-based platforms (chatbots, summarisation tools, AI overviews, etc.) were explicitly prohibited during the conventional learning condition. These materials reflect typical self-directed study practices in UK medical schools, where students revise using textbooks, summary notes, and non-AI online searches. This setup was chosen to represent a realistic and practical learning environment, allowing a fair comparison with the AI-supported condition.

#### 2.3.2. LLM Chatbot: Lenny AI

**Figure 1:**
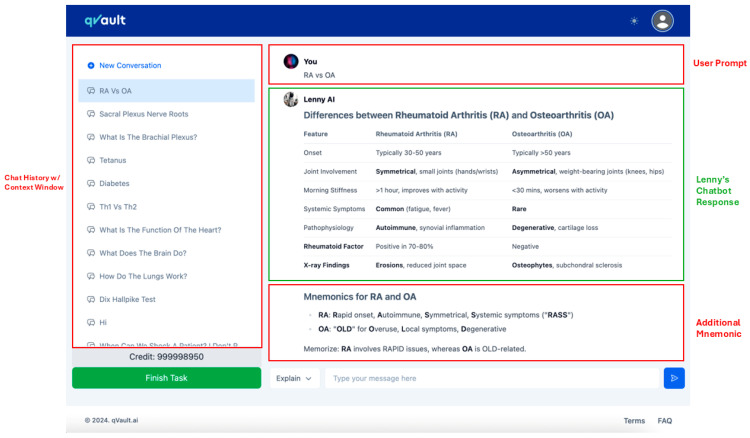
User interface layout of qVault.ai’s LLM chatbot “Lenny AI” during a medical query session.

The intervention group used Lenny AI, a custom-designed educational chatbot developed by the qVault team, built on the ChatGPT-4o LLM created by OpenAI (OpenAI, 2024; qVault.ai, 2025). Lenny AI was created to simulate a domain-specific teaching assistant tailored to the UK undergraduate medical curriculum. It provides text-based, interactive responses to typed user queries, focusing on clinically oriented anatomy for this study. The chatbot was hosted on a secure, web-based interface and made accessible only to study participants during the experimental period. To demonstrate, the figure below shows the structured user interface of Lenny AI learning platform (Figure 1). The left panel (outlined in red) shows the chat history and context window, listing previously asked questions. The top section contains the user prompt; the main chat window (outlined in green) presents Lenny AI’s response, and below the main response, an additional mnemonic section (outlined in red) summarises the comparison using memory aids.

Lenny AI is not a Retrieval-Augmented Generation (RAG) system, nor is it fine-tuned on proprietary or external data (Lewis et al., 2021). Instead, its outputs were generated directly from the base GPT-4o model, guided by robust prompt engineering and custom runtime configurations. These included a temperature setting of 0.3, an input token cap of 300, and an output limit of 1000 tokens, all chosen to balance fluency with factual reliability and maintain a concise, high-yield interaction style. Sampling parameters were calibrated to suppress generative randomness while retaining pedagogical flexibility. The system operated under a set of instructional guardrails that shaped output formatting and reasoning style. Prompts directed the model to employ formal medical terminology; each response is limited to approximately 150 words to ensure reading efficiency, conciseness, and presenting information in structured layouts such as tables and lists. Each answer includes a 30-word section with tailored mnemonics to support cognitive retention. These constraints were designed to mimic institutional relevance without requiring dynamic integration with local lecture content. To assess the reliability of Lenny AI’s outputs, the research team conducted structured internal trial sessions prior to participant access, using identical prompts, model configurations, and task formats as those planned for the study. These sessions replicated realistic student interactions under real-time conditions. Chatbot responses were qualitatively reviewed by team members with clinical and pedagogical expertise to evaluate factual accuracy, clinical appropriateness, clarity, and alignment with established medical references. While occasional minor ambiguities or slight imprecisions were noted, no clinically inaccurate or unsafe content was identified. Given the consistent behaviour demonstrated during these sessions, and supported by tightly controlled prompting and conservative generation parameters (e.g., low temperature), the team determined that Lenny AI met an appropriate standard of reliability for use in this educational research context. Our validation approach provided confidence that participants interacted with an appropriately vetted tool, while acknowledging the inherent variability associated with real-time generative models, as actual student-generated prompts may occasionally produce outputs not identified in pre-study simulations.

It is worth noting that, although this paper evaluates a specific tool, the implications extend beyond Lenny AI itself. The chatbot was internally reviewed and validated by the research team for medical relevance and content accuracy prior to deployment. Given that this implementation represents a high-performing, instruction-optimised use of an LLM, it serves as a conservative benchmark. If a custom-built, pedagogically structured chatbot demonstrates cognitive, epistemic, or performance-related limitations, then such issues are likely to be more pronounced in generic or commercially unrefined systems. At the same time, certain limitations were observed in this study, particularly those related to source transparency, curriculum alignment, and reasoning depth. Lenny AI operates within the constraints of its foundational GPT-4o training data, which, while comprehensive, may lack alignment with local curricula. These limitations may potentially be mitigated through the incorporation of RAG frameworks or reasoning-optimised architectures in future iterations. As such, the findings offer both a diagnosis of current constraints and a direction of travel for future chatbot development in medical education.

### 2.4. Study Procedures

#### 2.4.1. Task 0: Baseline AI Perception Assessment

At the beginning of the session, participants received a brief orientation outlining the study rationale and were introduced to Lenny AI. To establish baseline familiarity and attitudes toward AI, participants completed a pre-study AI literacy and perception questionnaire. This questionnaire, comprising 20 items and administered via Google Forms (see Appendix A), was distributed immediately prior to the academic tasks. In this context, baseline refers to participants’ existing familiarity with and attitudes toward AI (as assessed in task 0), used as a reference point for comparing changes observed later in the study.

#### 2.4.2. Task 1 and Task 2: Randomised Crossover Academic Tasks

Participants were randomly assigned a number between 001 and 020 using an online random number generator. Allocation to study arms was determined by number parity: odd-numbered participants (Arm 1) began with the LLM chatbot condition, while even-numbered participants (Arm 2) began with conventional study tools.

**Figure 2:**
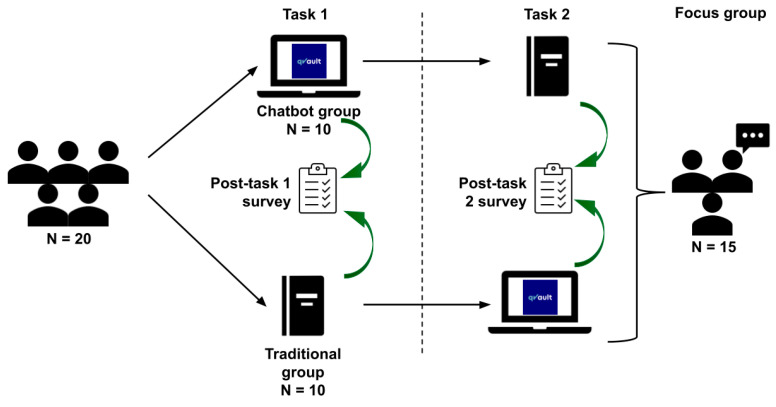
LLM chatbot randomised crossover study design. All 20 participants completed two academic tasks (10 SBAs and 6–7 unscored SAQs each), with 20 min per task and a 10 min break in between. In Task 1, Arm 1 (*n* = 10) used the LLM chatbot (qVault.ai), while Arm 2 (*n* = 10) used conventional resources (printed textbooks, non-AI web search). Arms then crossed for Task 2. Post-task surveys followed each task. Fifteen participants joined an optional focus group after both tasks.

Each group completed Task 1 under their assigned condition, followed by a 10 min break and a crossover: Arm 1 proceeded to conventional materials, while Arm 2 transitioned to the LLM chatbot for Task 2. Each academic task was time-limited to 20 min to standardise cognitive load and reduce variability in task exposure. This randomised crossover design aimed to minimise inter-cohort variability and control for participant-level confounders (Figure 2).

Each academic task included 10 SBA questions and 6–7 Short Answer Questions (SAQs), all mapped to the KCL Medicine MBBS curriculum. Participants were given 20 min per task. Task 1 focused on the anatomy and clinical application of the brachial plexus, while Task 2 addressed the lumbosacral plexus. Although both question sets were designed for preclinical students, they incorporated structured clinical vignettes to holistically assess applied anatomical knowledge and early interpretive reasoning (see Appendix B and Appendix C).

#### 2.4.3. Post-Task Questionnaire

Following each academic task, participants completed post-task questionnaires via Google Forms (see Appendix D and Appendix E), assessing their perceptions of the learning method used in that task. The first and second questionnaires included 18 and 22 items, respectively, using 5-point Likert-type scales to capture agreement with statements across multiple domains of perceived learning efficacy, usability, and engagement (Likert, 1932). The second questionnaire included additional exploratory items designed to capture broader aspects of the user experience. While only a subset of these items was used in the primary analysis, the extended format allowed for more comprehensive feedback and may support secondary analyses in future work.

#### 2.4.4. Focus Group Discussion

After completing both learning conditions, 15 of the 20 participants voluntarily joined post-task focus group discussions to further explore their experiences with the LLM chatbot and conventional study materials. Three focus groups with 5 participants each were facilitated by two hosts and one transcriber over two sittings (2 groups on Day 1 and 1 group on Day 2), with each discussion lasting approximately 30 min.

Discussion topics were structured around nine core domains:

1. Experience with AI
2. Changes in perceptions of AI
3. Comparative effectiveness of AI tools
4. Impact of AI on learning
5. Usability and engagement with AI
6. Challenges in using AI
7. Potential future influences of AI
8. Perceived role of AI in medical education
9. Suggestions for improving Lenny AI

Real-time transcription was conducted by the facilitator, supplemented by contemporaneous field notes to ensure completeness. These transcripts were subsequently used for thematic analysis (Braun & Clarke, 2006). (see Appendix F).

### 2.5. Blinding and Data Anonymisation

Due to the interactive nature of the intervention, participants were aware of the learning method used in each task. However, all data analysis was conducted in a blinded manner. Questionnaire responses and qualitative transcripts were anonymised prior to statistical processing to reduce the potential for researcher bias.

### 2.6. Data Analysis

#### Quantitative Analysis

Baseline characteristics were summarised descriptively using spreadsheet formulae. To assess within-subject differences in perception between Task 1 and Task 2, the distribution of change scores was evaluated using the Shapiro–Wilk test, which indicated non-normality, likely attributable to the sample size of 20 (Shapiro & Wilk, 1965). Accordingly, the non-parametric Wilcoxon signed-rank test was used to compare paired responses, with statistical significance defined as *p*-value < 0.05 (Wilcoxon, 1945). Due to the non-parametric nature of the analysis and the small sample size, a formal power calculation was not feasible. However, the crossover design improves statistical efficiency by controlling for inter-individual variability.

Performance scores were calculated as the percentage of correct responses on the 10 SBA items in each task. Four comparisons were performed:

1. Between-arm performance in Task 1 (Lenny AI vs. conventional tools)
2. Between-arm performance in Task 2 (conventional tools vs. Lenny AI)
3. Within-arm performance change in Arm 1 (Lenny AI → conventional tools)
4. Within-arm performance change in Arm 2 (conventional tools → Lenny AI)

As performance scores conformed to a normal distribution (based on Shapiro–Wilk testing), these comparisons were conducted using *t*-tests.

To examine the association between performance outcomes and participant perceptions, Spearman’s rank correlation coefficients were calculated between the percentage scores and each of the 12 perception metrics (Spearman, 1904) (see Table 1). Where significant associations were observed, follow-up Mann–Whitney U tests were used to compare perception scores between learning conditions (Mann & Whitney, 1947). This supplementary analysis aimed to identify whether the use of the LLM chatbot modified the relationship between perceived and measured learning performance.

There were no participant dropouts during the study, which was conducted over two days over two sittings. One missing response was recorded for the “ease of use” item in the baseline perception questionnaire. This data point was excluded from the analysis of that variable, with all other responses retained.

### 2.7. Qualitative Analysis

Focus group discussions were conducted using a semi-structured question guide designed to elicit participants’ views on learning efficacy, usability, perceived credibility, and future integration of AI tools in medical education. The guide included eight core questions, each with optional follow-up prompts, covering domains such as engagement, critical thinking, and comparative perceptions of learning methods. The full question set is provided in Appendix G.

Transcripts were then subjected to thematic analysis. Three independent coders reviewed the data and extracted representative quotations, which were then categorised by theme. Inter-rater reliability was assessed using Cohen’s Kappa coefficient, calculated pairwise between each coder dyad (rater 1 vs. rater 2, rater 2 vs. rater 3, rater 1 vs. rater 3) to account for the method’s assumption of two-rater comparisons (Cohen, 1960). All analyses were conducted using IBM Statistical Package for Social Sciences (SPSS) statistical software (version 30.0.0) (IBM, 2025).

The perception questionnaire was adapted from previously validated instruments in the domains of AI education and technology acceptance (Attewell, 2024; Malmström et al., 2023), with modifications made to suit the medical education context and align with the study’s objectives.

## 3. Results

Among the 20 participants, 13 (65%) identified as female and 7 (35%) as male. The mean age was 19.05 years (SD = 1.47), with a range of 18 to 24 years. 16 participants (80%) reported prior experience using chatbots; 4 (20%) had no previous exposure.

### 3.1. Baseline Perceptions

Prior to the intervention (Task 0), participants expressed moderate confidence in their ability to use LLM chatbots effectively (M = 3.05, SD = 1.00). Confidence in applying information derived from LLMs was similar (M = 2.95, SD = 1.00). Perceived usefulness of AI tools was rated as highly helpful (M = 4.32, SD = 0.75), while ease of use was also positively rated (M = 3.79, SD = 0.79). However, perceived accuracy of chatbot-generated responses was more moderate (M = 3.50, SD = 0.89), indicating some initial scepticism.

Participants rated chatbots moderately in their ability to support critical thinking (M = 3.40, SD = 0.88), but more favourably in terms of saving time (M = 3.70, SD = 1.17). Concerns regarding academic integrity were low (M = 2.10, SD = 1.02). There was strong agreement that AI will play a significant role in the future of medical education (M = 4.17, SD = 0.71), and participants expressed a high likelihood of using LLM chatbots in future studies (M = 4.20, SD = 0.83). Perceived importance of AI literacy was more moderate (M = 3.35, SD = 0.99).

### 3.2. Quantitative Findings

**Hypothesis** **1:** *Participants Will Report Significantly Higher Scores in the Measured Perception Parameters When Using the LLM Chatbot Compared to Conventional Study Tools.*

Twelve outcome domains were analysed from the post-task questionnaires based on a 5-point Likert scale: ease of use, satisfaction, efficiency, confidence in application, information quality, information accuracy, depth of content, ease of understanding, engagement, critical thinking, perceived performance compared to usual methods, and likelihood of future use. Analyses were limited to within-subject comparisons (i.e., Task 1 vs. Task 2) for each study arm independently. Results are summarised below and presented in Table 2.

Across all twelve outcome domains, no statistically significant differences were identified in favour of conventional tools over the LLM chatbot. On the other hand, five domains showed consistent and statistically significant preference for the LLM chatbot over conventional tools across both arms: ease of use, satisfaction, ease of understanding, engagement, and perceived quality of information.

- Ease of Use: Participants rated the chatbot as significantly easier to use than traditional materials (Arm 1: Mean Difference (MD) = 1.40, *p* = 0.040; Arm 2: MD = 1.20, *p* = 0.030). However, this difference did not reach statistical significance when compared with baseline expectations (Arm 1: *p* = 0.170; Arm 2: *p* = 0.510).
- Satisfaction: Satisfaction scores were significantly higher in the chatbot condition (Arm 1: MD = 1.40, *p* = 0.030; Arm 2: MD = 1.10, *p* = 0.037).
- Quality of Information: Both arms rated the chatbot more highly in terms of information quality (Arm 1: MD = 1.20, *p* = 0.050; Arm 2: MD = 1.00, *p* = 0.050). Notably, Arm 1 participants reported a significant improvement in their perception of information quality from baseline (3.40 to 4.30; *p* = 0.020)
- Ease of Understanding: The chatbot condition was rated more favourably in terms of ease of understanding, where both arms reported a higher score for the question “How easy was it to understand the information provided by your given learning method?” (Arm 1 MD = 1.30; Arm 2 MD = 1.40; both *p* = 0.010)
- Engagement: Chatbot use was associated with significantly higher engagement scores (Arm 1 MD = 1.60, *p* = 0.010; Arm 2 MD = 1.50, *p* = 0.005).

While several domains showed overall preference for the chatbot, some outcomes demonstrated statistically significant differences only within one study arm.

- Efficiency:
  ○ Arm 1 (chatbot-first) reported significantly greater perceived efficiency (MD = 1.70, 4.40 vs. 2.70; *p* = 0.020) whilst completing Task 1.
  ○ Arm 2 (conventional-first) showed no significant change (MD = 0.60, 3.60 vs. 3.00; *p* = 0.220).
- Confidence in Applying Information:
  ○ Arm 1: Participants felt significantly more confident applying information learned from the chatbot (MD = 0.90, 3.40 vs. 2.5; *p* = 0.020).
  ○ Arm 2: The increase was smaller and did not reach statistical significance (MD = 0.80, 3.30 vs. 2.50; *p* = 0.060).
- Perceived Performance Compared to Usual Methods:
  ○ Arm 1: The difference was not statistically significant (MD = 0.80; *p* = 0.110).
  ○ Arm 2: Participants reported a significant increase in perceived performance using the chatbot (MD = 1.00, 3.50 vs. 2.50; *p* = 0.040).
- Likelihood of Future Use:
  ○ Arm 1: Reported a significantly greater intention to use chatbots in future learning (MD = 1.20; *p* = 0.020).
  ○ Arm 2: The increase approached significance (MD = 0.90; *p* = 0.060).

Across the remaining outcome domains, results were more variable and, in some cases, non-significant.

In Arm 2, there was a statistically significant increase in participants’ perceptions of the accuracy of information provided by the chatbot following Task 2, relative to Task 0 (M = 3.30 to 4.20; MD = 0.90, *p* = 0.046). No significant change was observed in Arm 1. This suggests a possible effect of direct engagement on perceived information reliability.

Perceptions of content depth and critical thinking did not differ significantly between learning methods in either arm. For depth of content, Arm 1 approached statistical significance (MD = 1.30, *p* = 0.060), while Arm 2 showed a smaller, non-significant effect (MD = 0.80, *p* = 0.160). Similarly, ratings for critical thinking did not yield meaningful differences (Arm 1: MD = 1.10, *p* = 0.120; Arm 2: MD = 0.30, *p* = 0.520).

**Hypothesis** **2:** *The Use of the LLM Chatbot Will Result in Higher SBA Performance Scores Compared to Conventional Tools.*

Objective performance, measured via percentage scores on SBA questions, did not differ significantly across arms or tasks (Table 3).

In Task 1, participants in Arm 1 (chatbot-first) achieved a mean score of 71.43% (SD = 15.06), compared to 54.29% (SD = 23.13) in Arm 2 (conventional-first), yielding a mean difference (MD) of 17.14% (95% CI: −1.20 to 35.48; *p* = 0.065). In Task 2, where the arms were reversed, Arm 2 (chatbot) scored 63.33% (SD = 18.92) and Arm 1 (conventional) scored 68.33% (SD = 26.59), with a non-significant MD of –5.00% (95% CI: −16.68 to 26.68; *p* = 0.634).

Within-arm comparisons yielded similarly non-significant findings. In Arm 1, performance decreased slightly between Task 1 and Task 2 (MD = −3.10%; 95% CI: −15.41 to 21.60; *p* = 0.7139). In Arm 2, performance increased from 54.29% to 63.33% (MD = 4.99%; 95% CI: −23.09 to 9.04; *p* = 0.179).

**Hypothesis** **3:** *Perception Scores from Participants Using the LLM Chatbot Correlate with Performance Scores.*

**Figure 3:**
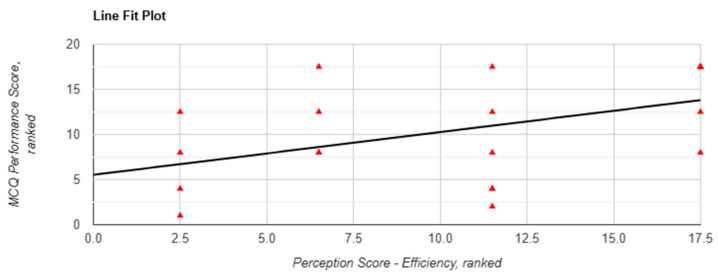
Correlation between perceived efficiency and MCQ performance scores in task 1 when using Lenny AI chatbot. Each red triangle represents a participant’s ranking: perception score (X-axis) and performance score (Y-axis) among 20 participants. Tied scores were assigned the same rank.

**Figure 4:**
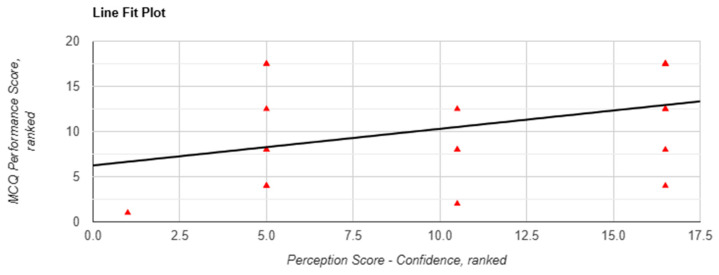
Correlation between perceived confidence in applying information and MCQ performance scores in task 1 when using Lenny AI chatbot. Each red triangle represents a participant’s ranking: perception score (X-axis) and performance score (Y-axis) among 20 participants. Tied scores were assigned the same rank.

Although absolute performance did not vary significantly, correlation analyses revealed associations between subjective perception and performance, particularly in Task 1. Perceived efficiency was significantly correlated with performance (r(18) = 0.469, *p* = 0.037, Figure 3), and the Mann–Whitney U test showed a significant between-arm difference favouring chatbot use (*p* = 0.004). Confidence in applying information was associated with a nonsignificant positive correlation with performance (r(18) = 0.392, *p* = 0.087, Figure 4) and was significantly higher in the chatbot group (*p* = 0.049).

**Figure 5:**
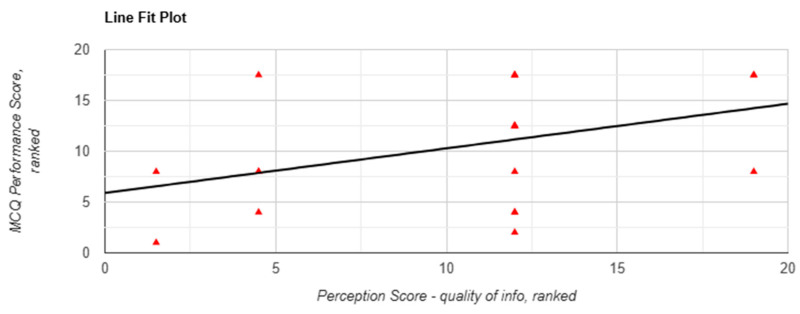
Correlation between perceived quality of info and MCQ performance scores in task 1 when using Lenny AI chatbot. Each red triangle represents a participant’s ranking: perception score (X-axis) and performance score (Y-axis) among 20 participants. Tied scores were assigned the same rank.

**Figure 6:**
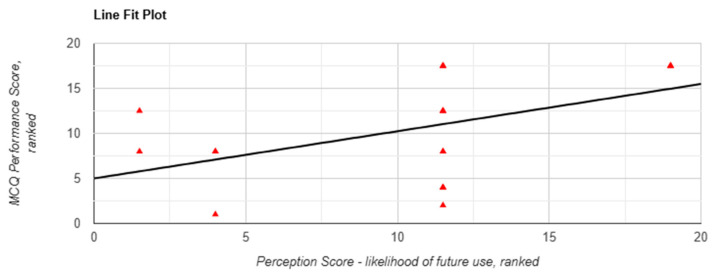
Correlation between likelihood of future use and MCQ performance scores in task 1 when using Lenny AI chatbot. Each red triangle represents a participant’s ranking: perception score (X-axis) and performance score (Y-axis) among 20 participants. Tied scores were assigned the same rank. No significant correlations were observed between performance and any perception measures in Task 2, suggesting that the strength of association may vary by exposure order or content domain.

A similar trend was observed for perceived quality of information, which was non-significantly correlated with performance (r(18) = 0.409, *p* = 0.073, Figure 5), but the corresponding Mann–Whitney U test yielded a statistically significant result in favour of the chatbot group (*p* = 0.003). Likelihood of future use showed a significant correlation (r(18) = 0.475, *p* = 0.034, Figure 6), although the between-arm difference was not significant (*p* = 0.214).

### 3.3. Thematic Analysis

**Figure 7:**
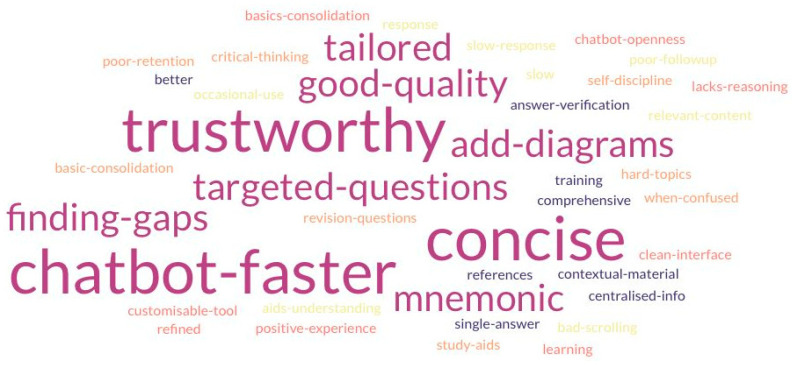
Word cloud of phrases cited by participants during focus group discussions.

Qualitative insights were drawn from focus group transcripts, thematically analysed by three independent coders. Twelve key themes were identified, reflecting both the perceived benefits and limitations of LLM chatbot use in medical education (Table 4). Inter-rater agreement ranged from fair to substantial (Cohen’s κ = 0.403–0.633), indicating acceptable reliability of thematic classification. A third-coder adjudication step was not feasible due to resource constraints. Instead, all disagreements were resolved through discussion to achieve a final consensus. The themes are identified and separated into two categories, and the key phrases are represented in the word cloud (Figure 7), where the size of each phrase reflects the frequency with which it was mentioned across all participants. Key perceived strengths of the chatbot included its trustworthiness, speed, and conciseness. The most frequently suggested area for improvement was the integration of visual aids, such as diagram generation.

#### 3.3.1. Speed and Efficiency

Participants consistently identified speed as a primary advantage of chatbot use. The tool was seen as particularly effective for rapid information retrieval and clarification of discrete medical queries. This conciseness was particularly beneficial when students needed quick clarification or a general topic overview. One participant noted that “if it was a single answer, then the chatbot was better” than conventional sources. Others contrasted this with conventional methods, which required “a lot longer to filter through information”.

#### 3.3.2. Depth and Complexity

While chatbots were viewed as efficient, several participants expressed concern about limitations in the depth of explanation and conceptual scaffolding. Conventional study methods were regarded as more comprehensive for building foundational understanding and exposure to broader discussions of the inquiry, with one student commenting that “traditional [conventional methods] gave residual information useful for understanding”. Others felt the chatbot offered less engagement and limited support for deeper learning, with one remarking that “Googling and using notes enhanced critical thinking instead of [using] the chatbot”.

#### 3.3.3. Functional Use Case and Focused Questions

The chatbot was seen as effective for addressing specific knowledge gaps, but less suited for comprehensive topic review. Several participants reported the chatbot answering direct questions better and using it to reinforce rather than initiate learning: “Chatbot is better for specific questions” and “more useful with a specific query in mind instead of learning [an] entire topic”. Concerns were also raised about knowledge retention, with one stating, “didn’t allow retaining the information” and another, “Better for consolidating already learnt basic knowledge”. This statement positions the chatbot less as a teacher, and more as the academic equivalent of a highlighter pen: useful, but only if you already know what’s important.”

#### 3.3.4. Accuracy and Credibility

Perceptions of chatbot accuracy were mixed. While most participants were positively surprised by the reliability of AI-generated content (e.g., “was surprised to use a chatbot for reputable information”), some emphasised the need to corroborate responses with trusted academic sources. There were repeated suggestions to improve credibility by incorporating references: “more useful if references are included in chatbot responses” and “will trust ChatGPT more if it is trained based on past papers”.

#### 3.3.5. Openness to AI as a Learning Tool

Most participants expressed openness to using LLM chatbots as supplementary learning tools. One stated, “more open to using chatbots after this [study]”. However, there was widespread agreement that chatbots should not supplant textbooks or peer-reviewed material. A participant summarised this sentiment: “In its current state, I would only use it from time to time”.

#### 3.3.6. Curriculum Fit

Students frequently noted a disconnect between chatbot content and their specific medical curriculum, requiring additional effort to contextualise the information. The AI’s output was seen as generic and occasionally misaligned with institutional learning outcomes. One participant suggested: “best to train it to be tailored to [the] curriculum to ensure relevance”, hinting at further developments, such as utilising Retrieval Augmented Generation (RAG) techniques to ensure alignment. Another proposed that better questions could be generated if tailored to the uploaded lecture content. This suggestion reflects not only the desire for personalisation but also the implicit truth every student learns early: if it is not on the syllabus, it might as well be wrong.

#### 3.3.7. Further Development and Technical Limitations

Overall, the chatbot’s interface received positive feedback. One participant noted, “The UI (User Interface) is very clean and easy to use”, suggesting that a smooth user experience and design played a key role in its usability. However, some participants encountered usability challenges that impacted their experience. One participant noted that “Scrolling to the bottom wasn’t smooth”. Participants noted latency issues, mentioning that the chatbot takes too long to generate responses and could deter them from using the chatbot. They “tend to Google it instead if it takes too long” and “Delay could be frustrating”.

These limitations highlight the need for further development to improve user experience and content delivery. Suggestions for future development included the addition of diagrams, better mnemonic aids, and interactive learning tools: “generate Ankis, questions, and diagrams from PowerPoint”.

## 4. Discussion

This study employed a mixed-methods, crossover design to examine the pedagogical value of LLM chatbots in undergraduate medical education. By integrating quantitative data with qualitative insights, the findings offer a nuanced understanding of how AI tools influence learning processes. While participants consistently reported improvements in usability, efficiency, and engagement, these benefits appeared to come at the expense of cognitive depth and integrative understanding. It is important to note that participants were novice Year 1 medical students, and findings should be interpreted in light of their early stage of professional development.

A further methodological consideration relates to the recruitment strategy via posters, which may have introduced a degree of self-selection bias. This approach is more likely to attract students with an existing interest or curiosity about educational technologies, potentially skewing the sample toward individuals with more favourable attitudes toward AI tools. While this limits the generalisability of the findings to the broader student population, it remains consistent with the study’s focus on understanding the experiences of actively engaged users.

Before interpreting the findings, it is also important to address the ecological validity of the study’s design. We aimed to evaluate chatbot-supported learning under constraints that authentically mirror summative assessment in preclinical anatomy, such as time-limited Single Best Answer (SBA) tasks. For the crucial context of exam preparation, this represents a realistic ecology. A fully naturalistic setting, while different, would have introduced uncontrolled variables (such as time-on-task, concurrent resources, and interruptions) that would diminish the causal interpretability sought through the randomised crossover design. We therefore consider the present setting to be not only appropriate for the study aim but also ecologically valid for an assessment-aligned study.

Our four hypotheses were variably supported. In line with the Technology Acceptance Model (1), students rated the chatbot higher for usability, satisfaction, and engagement. Cognitive Load Theory (2) was partially supported: efficiency and clarity improved, but depth and critical thinking did not. Dual-Process Theory (3) explained this imbalance, with chatbot use privileging rapid recall (System 1) but not reflective reasoning (System 2). Finally, performance outcomes (4) did not differ significantly between groups, although positive correlations between perceptions (e.g., efficiency, confidence) and SBA scores suggest potential underpowered effects. These hypotheses are explored in greater depth in the thematic sections below.

### 4.1. The Efficiency-Depth Paradox: When Speed Compromises Comprehension

A central finding concerns what may be termed the efficiency-depth paradox. Participants found the chatbot to be significantly easier to use than conventional materials, with higher ratings for satisfaction, engagement, and perceived information quality. These improvements were supported by both statistical analysis and thematic feedback, with students praising the tool’s speed and conciseness. However, measures of content depth and critical thinking did not improve significantly, and student feedback frequently reflected concern about superficiality. As one participant noted, the chatbot was “more useful with specific queries” but lacked the capacity to “show how everything is related”. Depth of content—a key measure of how well students engage with, contextualise, and interrelate information—did not exhibit meaningful improvements. Participants nevertheless rated the chatbot markedly higher for ease of use, satisfaction, and engagement. These outcomes align closely with TAM’s dimensions of perceived ease of use and perceived usefulness, indicating that the model effectively accounts for the strong appeal of the tool despite its limited capacity to foster deeper cognitive engagement.

This observed tension may tentatively be interpreted through Cognitive Load Theory (CLT). LLM chatbots may help reduce extraneous cognitive load (the mental effort imposed by irrelevant or poorly structured information) by streamlining access to targeted information, which could be particularly beneficial in time-constrained learning environments such as medicine. However, minimising extraneous load does not automatically increase germane cognitive load, defined as the effort devoted to constructing and integrating knowledge structures (Sweller, 2011; Gualda-Gea et al., 2025). In this small sample, although participants reported higher efficiency and ease of understanding, this did not appear to translate into deeper learning outcomes, which may suggest limited activation of the cognitive processes needed for long-term retention and schema development. That said, for learners with less developed metacognitive strategies, such as difficulty with content triage, synthesis, or task regulation, the chatbot could potentially function as a cognitive scaffold. By mitigating surface-level overload, it might enable more efficient resource allocation toward germane cognitive processes than would typically be achievable with conventional, unstructured materials. In such cases, the chatbot does not merely streamline information retrieval but actively supports a more stable cognitive load distribution, thereby facilitating more sustained engagement. Although this interpretation remains speculative given the limited sample size, it provides a possible explanation for the heterogeneous patterns observed across both performance outcomes and user perceptions. Longitudinal studies tracking learners’ cognitive development over time would provide more definitive insights into how AI tools influence schema construction and knowledge integration.

The Dual-Process Theory may offer further explanatory insight (Evans & Stanovich, 2013). In our study, the custom-built Lenny chatbot appeared to predominantly support outcomes consistent with System 1 cognition—fast, intuitive, and suitable for factual recall (Croskerry, 2009). Yet deeper conceptual learning in medicine depends on System 2 cognition—deliberate, reflective, and analytical (Pelaccia et al., 2011). Given the lack of observed improvement in critical thinking domains and depth of content, this imbalance in cognitive processing is noteworthy. Participant feedback reinforced the distinction: while Lenny facilitated quick answers, it did not consistently prompt reflective engagement and often felt transactional or superficial. In other words, even a tailored, curriculum-aligned chatbot seemed to fast-track learners down a highway, occasionally bypassing the scenic route of reflective reasoning. This suggests that the surface-deep gap may persist across both domain-specific and general-purpose chatbots (Marton & Säljö, 1976; Biggs & Tang, 2011; Lucas et al., 2024; Arun et al., 2024), unless scaffolding and task design are explicitly directed toward deeper engagement (Oyler & Romanelli, 2014; Ho et al., 2023). Recent releases such as ChatGPT’s “Study Mode,” which incorporates Socratic prompting and adaptive questioning, appear to respond directly to these limitations (OpenAI, 2025). The timeliness of our findings highlights the need for empirical evaluation of such pedagogical innovations in medical education. LLM chatbots, if unmodified, have the potential to reinforce surface learning strategies at the expense of higher-order thinking. To mitigate this, chatbot design might benefit from incorporating adaptive scaffolding—for instance, requiring learners to articulate reasoning or engage in structured reflection before receiving answers. Such strategies could help encourage transitions from intuitive to analytical processing, aligning the tool more closely with deep learning objectives. However, scaffolding should support clarity without displacing opportunities for self-directed reasoning.

Finally, these limitations also articulate the importance of pedagogical complementarity. AI tools are perhaps best used to augment, not replace, methods that foster dialogue, exploration, and self-reflection. For example, chatbots may be particularly useful in supporting flipped classroom models or hybrid learning strategies, in which they serve as preliminary tools for foundational knowledge acquisition, followed by an in-person, case-based discussion to promote deeper conceptual engagement and potentially mitigate the trade-off.

### 4.2. Confidence Versus Competence: The Illusion of Mastery

This small-scale study revealed a potential dissociation between students’ self-reported confidence and their demonstrated cognitive performance. The consistently elevated scores for usability, satisfaction, and engagement further reflect TAM’s two central constructs, perceived ease of use and perceived usefulness. This theoretical framing helps explain why students reported feeling more confident when working with the chatbot, even though their actual competence, as reflected in SBA performance, did not significantly improve. Participants exposed to the LLM chatbot reported significantly greater confidence in applying information, alongside improved perceptions of information accuracy. However, these perceptions were not accompanied by measurable improvements in critical thinking or consistent gains in academic performance. In some instances, performance declined relative to conventional methods. Despite this disconnect, participants expressed a strong intention to continue using the chatbot. This may suggest high user endorsement, yet also increases the possibility of overestimating one’s mastery based on the immediacy and clarity of AI responses.

Qualitative feedback reinforced this discrepancy. Many students viewed the chatbot as a confidence-boosting tool, frequently citing its clarity, speed, and directness. Several commented that its concise and unambiguous format made information feel more accessible than conventional materials, reducing uncertainty when studying. However, others voiced concern that this simplification limited deeper engagement, describing the chatbot as helpful for rapid fact-checking but insufficient for promoting reflective or analytical thinking.

These findings align with the Technology Acceptance Model (TAM), which posits that perceived ease of use and perceived usefulness drive user adoption (Davis, 1989). In our data, the chatbot was consistently rated higher for ease of use, satisfaction, engagement, and perceived quality, which are direct indicators of TAM’s two core constructs. Functionality was therefore mapped directly onto these TAM dimensions, helping explain the uniformly positive user experience even in the absence of consistent performance gains. Yet TAM does not imply that usability ensures deep cognitive engagement. While the chatbot’s streamlined interface may have facilitated recall and fluency, it offered little scaffolding for critical thinking or integrative reasoning. This distinction is important: fluency can be misinterpreted as understanding, fostering cognitive overconfidence where students feel assured without achieving conceptual mastery. The educational implications, while preliminary, could be meaningful. While confidence enhances engagement and can motivate further learning, confidence without competence poses risks, particularly in clinical education, where overconfidence may translate into diagnostic error. AI-based learning tools should therefore be designed to temper misplaced certainty and mitigate overconfidence, ensuring that learners interrogate their understanding rather than accept fluency as a proxy for insight.

One potential solution lies in instructional scaffolding embedded within chatbot interactions. For example, prompting students to articulate their reasoning before receiving answers may compel engagement with the underlying logic, fostering deeper processing. Similarly, adaptive AI systems could modulate task complexity based on expressed confidence, offering progressively challenging scenarios that test conceptual boundaries and guard against premature certainty. Future mediation analyses conducted on larger and more diverse samples (including students from other medical schools) could explore whether increases in self-reported confidence predict actual academic performance or if these effects reflect transient affective boosts without corresponding cognitive development.

### 4.3. Transparency and Traceability: The Foundations of Trust in AI Learning Tools

The perceived credibility of AI-driven learning tools may hinge not only on the accuracy of their outputs, but also on the transparency of their informational provenance. In our study, students reported significantly improved perceptions of the chatbot’s quality and accuracy over time, reflecting confidence in its technical performance. However, qualitative data also suggested ongoing concerns regarding the verifiability of responses and the absence of identifiable sources. This tension reflects a broader challenge in AI integration: how to foster epistemic trust in systems that deliver answers without evidentiary scaffolding.

Although the chatbot generally produced correct and relevant responses, many students hesitated to fully trust its outputs due to a lack of traceable citations or curricular alignment. Several explicitly requested embedded references and clearer links to validated educational materials. Its persona occasionally resembled an overenthusiastic peer: helpful, articulate, and entirely unreferenced. These student perspectives appear to align with current debates in algorithmic transparency, which emphasise that accuracy is insufficient without contextual legibility; that is, the ability of users to interrogate the epistemic basis of machine-generated outputs. In high-stakes educational settings such as medicine, where knowledge validity is paramount, tools that obscure their informational lineage may risk undermining their own utility.

TAM offers partial explanatory power here. While perceived ease of use and usefulness clearly facilitated chatbot adoption, our sample suggests that long-term engagement requires a deeper sense of control and visibility over system logic. Without transparency, students may rely on the chatbot for efficiency, but withhold full epistemic endorsement. In other words, accepting its outputs functionally, yet distrusting them academically.

This distinction is captured more precisely by epistemic trust theory, which holds that credibility depends on the perceived expertise, integrity, and openness of an information source (Origgi, 2004; McCraw, 2015; McMyler, 2011). In our findings, the chatbot met functional expectations of accuracy but fell short of epistemic credibility. Participants repeatedly described a desire for tools that did not merely appear accurate but enabled verification. Without mechanisms for students to trace, interrogate, and contextualise content, trust remained provisional.

Addressing this perceived credibility dilemma may require a dual-pronged approach. First, chatbots must maintain their efficiency and streamlined design, ensuring that they remain a highly accessible learning tool. Concurrently, it must incorporate mechanisms for transparency, allowing users to verify, interrogate, and expand upon the information provided. With these considerations in mind, the following design features are recommended:

- Citation Toggles: Allowing users to reveal underlying references where applicable, supporting source traceability.
- Uncertainty Indicators: Signalling lower-confidence outputs to prompt additional verification.
- Expandable Explanations: Offering tiered content depth, enabling students to shift from summary to substantiated detail on demand.

In the absence of these deeper structural redesigns, students may be more likely to remain selectively engaged with AI tools: turning to them for convenience, but withholding full epistemic reliance. It is worth noting, however, that superficial interface enhancements, such as citation toggles or confidence indicators, may elevate the appearance of trustworthiness but do little to guarantee its substance. This distinction is more than semantic; it is pedagogically fundamental. An interface that feels authoritative cannot compensate for outputs that remain unverifiable. The prioritisation of aesthetic fluency over evidentiary integrity in some AI-driven learning platforms may cultivate a form of functional trust that lacks epistemic depth, potentially leading to commercial success. Yet, in such cases, user experience becomes a surrogate for validation, offering a veneer of credibility while displacing the critical standards upon which educational authority must rest. Moreover, developing scalable, transparent trust mechanisms that meet both educational and epistemic standards remains a substantial design challenge that future AI systems will need to address, particularly if they are to be widely adopted in high-stakes learning environments.

Ultimately, trust in AI-assisted learning is not a function of fluency alone, but rather is built through transparency, traceability, and critical agency. Students must be empowered not only to accept chatbot-generated content, but to interrogate it, contextualise it, and, where appropriate, challenge it. Without such shifts in design, AI risks reinforcing passive consumption rather than fostering the critical appraisal skills essential to clinical education.

### 4.4. No Consistent Performance Gains from Chatbot Use

Although chatbot use did not yield statistically significant performance gains across the full sample, this absence of significance is itself a meaningful finding. In a field often characterised by projected growth and strong claims of effectiveness, these results offer a more cautious perspective, highlighting the importance of empirical testing over assumed benefit. Task-specific variation was observed: in Task 1, students using the chatbot outperformed peers using conventional resources, with reported efficiency, confidence, and information quality positively associated with performance. These effects were not seen in Task 2, and no consistent pattern emerged across tasks or study arms. While preliminary, relative performance improvements ranging from 5 to 17% may have practical value in time-limited learning contexts. This hypothesis merits further testing through larger, adequately powered studies. The use of engagement and learning analytics may also provide objective measures of cognitive load and long-term retention.

More cautiously, this inconsistency may indicate the need to move beyond the assumption of uniform benefit. Qualitative feedback reinforces this: students described the chatbot as most effective for discrete, fact-based tasks, while expressing limitations in areas requiring conceptual synthesis. Taken together, these findings suggest that the chatbot may favour learners who are already confident, goal-oriented, and proficient in self-directed learning, while offering less benefit to those who depend on scaffolding and structured reasoning to build understanding.

These observations may raise some questions about the adequacy of performance as a monolithic metric. Rather than relying solely on mean task scores, future studies should adopt more granular analytic approaches, such as mastery threshold models (e.g., via the Angoff method), residual gain analysis, or subgroup stratification based on learner confidence or cognitive style (Angoff, 1971). Where mastery thresholds are employed, triangulation with inter-rater reliability measures such as Cohen’s kappa would further strengthen methodological validity, particularly in larger cohorts (McHugh, 2012). Our findings tentatively indicate that AI tools may not equalise performance, but rather stratify it, reinforcing existing learner differences unless specifically designed to account for them.

Crucially, the absence of consistent performance gains should not be read as a failure of the chatbot per se, but as a call to rethink how such tools are designed and evaluated. Static delivery of content, regardless of how streamlined or accurate, is unlikely to yield uniform gains across diverse learner populations. AI tools must become more adaptive, sensitive to learner signals, task complexity, and evolving knowledge states. Incorporating diagnostic mechanisms that adjust the depth, format, or difficulty of chatbot responses based on real-time indicators of comprehension, while building on existing safeguards for content accuracy and transparency, could help bridge the gap between surface-level usability and meaningful educational value.

## 5. Limitations

While this study offers valuable preliminary insights into LLM chatbot use in medical education, several methodical considerations must be addressed in future work.

First, the modest sample size limits statistical power and increases susceptibility to Type II error, raising the possibility that some non-significant outcomes may reflect insufficient power rather than a true absence of effect. This is particularly relevant to domains such as depth of content and confidence, where medium-to-large effect sizes were observed but fell short of conventional thresholds. While several outcomes reached robust statistical significance with large effect sizes, future studies would benefit from larger samples and longer washout periods to improve internal validity and reduce the risk of both false negatives and spurious positives. In addition, applying correction procedures such as Bonferroni adjustment (Bonferroni, 1936) or false discovery rate control (Benjamini & Hochberg, 1995) can further safeguard against Type I error, though the primary concern in this pilot is the under-detection of potentially meaningful effects. Second, the study’s assessment was designed to move beyond simple factual recall. By utilising clinical vignettes, our SBA questions required participants to engage in application and analysis, which are higher-order skills within Bloom’s Taxonomy (Bloom, 1956). However, we acknowledge that even well-crafted SBAs may not fully capture the apex cognitive processes, such as synthesis and evaluation. To enhance ecological validity, future evaluations should incorporate performance-based assessments, such as a deep evaluation of SAQs, Objective Structured Clinical Examinations (OSCEs) or AI-integrated case simulations, which better reflect real-world diagnostic and decision-making demands (Messick, 1995; Van Der Vleuten & Schuwirth, 2005).

Third, while the experimental setting possesses high ecological validity for assessment-focused learning, its findings may not fully generalise to all forms of self-directed study. The controlled, task-oriented environment was a deliberate and necessary choice to maintain high internal validity and isolate the chatbot’s effect. However, this differs from more open-ended learning scenarios where students are not under the same time pressures. Future research could therefore explore the utility and pedagogical impact of AI chatbots in these more naturalistic learning environments to provide a more holistic understanding of their role in medical education.

Finally, the generalisability of LLM chatbot outputs remains constrained by the scope of their training data. If models are predominantly trained on Western biomedical literature and curricula, they may fail to accommodate context-specific variations in clinical practice, particularly in low- and middle-income countries (LMICs) (Whitehorn et al., 2021). This raises concerns regarding content validity, biases and the equitable applicability of AI tools across diverse educational systems. Moreover, our study focused solely on medical students, and it remains unclear whether findings would translate similarly to students from other disciplines with different learning needs, curricular structures, or baseline familiarity with AI tools. Future studies should explicitly assess the performance of chatbots across academic programs beyond medicine to understand broader applicability. In parallel, future work should also evaluate chatbot performance in non-Western curricular contexts, particularly those that prioritise competency-based learning and locally relevant clinical paradigms. A design-based research (DBR) approach may offer methodological advantages by enabling iterative refinement of chatbot features across real-world educational settings, thereby enhancing both practical relevance and theoretical insight (Brown, 1992).

## 6. Conclusions

This study offers early but compelling evidence that LLM chatbots can augment medical education by enhancing efficiency, engagement, and rapid information access. Yet these gains come with trade-offs. The limitations observed in fostering critical thinking, conceptual depth, and long-term retention reveal that chatbot use, while promising, is not pedagogically sufficient in isolation. Our findings advocate for a re-framing of AI not as a standalone tutor, but as a pedagogical partner. It should be best deployed within hybrid educational models that preserve the rigour of clinical training while leveraging the speed and accessibility of intelligent systems. The crossover design employed in this feasibility study serves as an early validation of student acceptance, building a robust foundation for larger-scale and longer-term evaluations.

Students consistently rated the chatbot higher than conventional resources across Technology Acceptance Model domains, including ease of use, satisfaction, engagement, and perceived information quality. This pattern accords with TAM’s central constructs of perceived ease of use and perceived usefulness, which together explain adoption intent and heightened confidence even when objective competence did not change.

From a Cognitive Load Theory perspective, perceived gains in efficiency and clarity indicate reductions in extraneous load through streamlined access to targeted content. The absence of measurable improvements in depth of content, critical thinking, or long-term retention indicates that germane load was not increased to support schema construction, which explains why surface fluency rose without parallel gains in integrative understanding.

Dual-Process accounts further clarify this dissociation: the chatbot experience appears to favour fast, intuitive retrieval and cue-driven problem-solving associated with System 1. Deeper conceptual learning in medicine depends on deliberate, reflective System 2 processes; students’ comments that the chatbot was better for specific questions but less helpful for connecting ideas reflect this imbalance between fluency and depth.

Finally, Epistemic Trust helps explain students’ ambivalence about accuracy and alignment. Functional trust in clarity and apparent correctness was high, yet the lack of references and explicit curricular anchoring constrained full epistemic endorsement. Designing for transparency and traceability is, therefore, necessary if AI tools are to command durable academic trust. Such scaffolding and transparency are best embedded within hybrid models that preserve rigour while integrating intelligent systems.

If AI is to earn its place in the future of medical education, it must not only deliver correct answers but also cultivate reasoning, reflection, and relevance. This is the line that separates educational technology from educational value and scalable access from scalable wisdom.

## Figures and Tables

**Table 1:** Outcome measures and corresponding survey questions.

Outcome Measures	Questions
Ease of Use	“How easy was it to use this learning method?”
Satisfaction	“Overall, how satisfied are you with this method for studying?”
Efficiency	“How efficient was this method in gathering info?”
Confidence in Applying Information	“How confident do you feel in applying the information learned?”
Quality of Information	“Rate the quality of the information provided.”
Accuracy of Information	“Was the information provided accurate?”
Depth of Content	“Describe the depth of content provided by the learning tool.”
Ease of Understanding	“Was the information easy to understand?”
Engagement	“How engaging was the learning method in maintaining your interest during the task?”
Performance Compared to Usual Methods	“Compared to usual study methods, how did this one perform?”
Critical Thinking	“How did this learning method affect your critical thinking?”
Likelihood of Future Use	“How likely are you to use this learning method again?”

**Table 2:** Baseline (T0) and post-task perception score differences across 12 domains for Arm 1 and Arm 2. A hyphen (-) indicates domains not assessed at baseline. Significant differences (* *p* < 0.050) are highlighted: green for both arms, yellow for one arm. SD = Standard Deviation. Chatbot use significantly improved scores in ease of use, perceived quality, understanding, and engagement in both arms. Efficiency, confidence, performance, and likelihood of future use improved in one arm only. Effect sizes were moderate to large for significant outcomes.

		Baseline (T0)		Perception Differences (T1 vs. T2)					
		Mean	SD	T1 Mean (SD)	T1 Median (Range)	T2 Mean (SD)	T2 Median (Range)	Effect Size (r)	*p* Value
Ease of Use	Arm 1	3.79	0.79	4.20 (0.92)	4.0 (3.0)	2.80 (0.79)	3.0 (2.0)	0.68	0.040 *
	Arm 2			3.00 (0.82)	3.0 (2.0)	4.20 (0.92)	4.5 (2.0)	0.75	0.030 *
Satisfaction	Arm 1	-	-	4.00 (0.94)	4.0 (3.0)	2.60 (1.16)	3.0 (3.0)	0.69	0.030 *
	Arm 2			2.70 (0.84)	3.0 (3.0)	3.80 (1.03)	4.0 (3.0)	0.73	0.040 *
Quality of information	Arm 1	3.4	0.68	4.30 (0.48)	5.0 (3.0)	3.10 (1.20)	2.5 (2.0)	0.75	0.050 *
	Arm 2			3.20 (0.79)	3.0 (2.00	4.20 (0.92)	4.0 (4.0)	0.75	0.050 *
Ease of Understanding	Arm 1	-	-	4.40 (0.97)	4.0 (2.0)	3.10 (0.88)	3.0 (3.0)	0.89	0.010 *
	Arm 2			3.00 (1.33)	2.0 (3.0)	4.40 (0.84)	3.0 (3.0)	0.88	0.010 *
Engagement	Arm 1	-	-	3.60 (0.97)	4.0 (1.0)	2.00 (0.82)	3.0 (4.0)	0.89	0.010 *
	Arm 2			2.70 (0.82)	3.0 (2.0)	4.20 (0.63)	4.5 (2.0)	0.89	0.005 *
Efficiency	Arm 1	-	-	4.40 (0.97)	4.0 (1.0)	2.70 (0.82)	4.5 (3.0)	0.72	0.020 *
	Arm 2			3.00 (0.82)	4.0 (2.0)	3.60 (1.17)	4.0 (2.0)	0.46	0.22
Confidence in applying information	Arm 1	3.05	1	3.40 (0.84)	4.0 (2.0)	2.50 (0.97)	2.5 (4.0)	0.9	0.020 *
	Arm 2			2.50 (0.97)	3.0 (2.0)	3.30 (1.06)	3.5 (3.0)	0.72	0.06
Performance compared to usual methods	Arm 1	3.3	0.86	3.40 (0.7)	5.0 (3.0)	2.60 (0.84)	3.0 (2.0)	0.56	0.11
	Arm 2			2.50 (0.97)	3.0 (4.0)	3.50 (0.85)	5.0 (2.0)	0.73	0.040 *
Likelihood of future use	Arm 1	3.25	0.97	4.00 (0.82)	3.5 (3.0)	2.80 (0.79)	2.0 (2.0)	0.75	0.020 *
	Arm 2			3.60 (0.84)	3.0 (3.0)	4.50 (0.71)	4.0 (2.0)	0.72	0.06
Accuracy of information	Arm 1	3.5	0.89	3.90 (0.32)	3.5 (2.0)	4.20 (1.03)	3.0 (3.0)	0.39	0.3
	Arm 2			3.90 (0.74)	3.0 (3.0)	4.20 (0.63)	3.5 (3.0)	0.37	0.41
Depth of content	Arm 1	-	-	4.20 (0.79)	4.0 (3.0)	2.90 (1.37)	2.5 (2.0)	0.59	0.06
	Arm 2			2.90 (0.74)	3.0 (2.0)	3.70 (1.06)	3.0 (3.0)	0.57	0.161
Critical thinking	Arm 1	3.4	0.97	3.70 (1.25)	4.0 (3.0)	2.60 (0.82)	3.0 (3.0)	0.55	0.12
	Arm 2			2.90 (0.85)	4.0 (3.0)	3.20 (0.79)	5.0 (2.0)	0.23	0.52

**Table 3:** Mean performance scores across study arms and tasks. This table summarises mean performance scores (percentage correct) for each study arm and task. No comparisons reached statistical significance. However, chatbot use in Task 1 was associated with a higher mean score compared to conventional tools. Within-arm differences between tasks were also non-significant, though trends favoured chatbot use. SD = Standard Deviation; CI = Confidence Interval.

Comparison	Task 1 Mean Score % (SD)	Task 2 Mean Score % (SD)	Mean Difference (%)	95% CI	*p*-Value
Task 1: Arm 1 vs. Arm 2	71.43 (15.06)	54.29 (23.13)	17.14	−1.20 to 35.48	0.065
Task 2: Arm 2 vs. Arm 1	63.33 (18.92)	68.33 (26.59)	−5	−16.68 to 26.68	0.634
Within Arm 1: Task 1 vs. Task 2	71.43 (15.06)	68.33 (26.59)	−3.1	−15.41 to 21.60	0.7139
Within Arm 2: Task 1 vs. Task 2	54.29 (23.13)	63.33 (18.92)	4.09	−23.09 to 9.04	0.179

**Table 4:** Themes related to chatbot ability and their associated features and functions. This table presents key themes and attributes identified through focus group analysis, highlighting perceived strengths (e.g., accuracy, speed, curriculum fit) and areas for improvement (e.g., technical limitations, further development) in the context of chatbot-assisted learning.

Ability	Features and Functions
Accuracy	Curriculum fit
Complexity	Focused questions
Credibility	Further development
Depth	Functional use case
Efficiency	Openness to AI as a learning tool
Speed	Technical limitations

## Data Availability

The data presented in this study are available on request from the corresponding author due to privacy restrictions.

## Abbreviations

The following abbreviations are used in this manuscript:

**Abbrev.**	**Full Form**
AKT	Applied Knowledge Test
AI	Artificial Intelligence
CI	Confidence Interval
CLT	Cognitive Load Theory
DBR	Design-Based Research
GDPR	General Data Protection Regulation
KCL	King’s College London
LLM(s)	Large Language Model(s)
LMIC(s)	Low- and Middle-Income Country(ies)
M	Mean
MD	Mean Difference
n	Sample size
OSCE(s)	Objective Structured Clinical Examination(s)
*p*	*p*-value
RAG	Retrieval-Augmented Generation
r	Correlation coefficient (effect size)
REMAS	Research Ethics Management Application System
SAQ(s)	Short Answer Question(s)
SBA	Single Best Answer
SD	Standard Deviation
SPSS	Statistical Package for the Social Sciences
TAM	Technology Acceptance Model
T0, T1, T2	Task 0 (baseline), Task 1, Task 2 timepoints
UI	User Interface
Z	Z-statistic
κ (kappa)	Cohen’s kappa coefficient

## Appendix A. Task 0 (Baseline) Questionnaire

What is your KCL email address (name@kcl.ac.uk)? Q1. What is your year group? A100 Year 1 EMDP Year 1A EMDP Year 1B A100 Year 2 EMDP Year 2 Other… Q2. Assigned ID number Q3. What is your age? Q4. What is your gender? Male Female Prefer not to say Other… Q5. Are you a local or international student? Local International Q6. Have you used AI LLM Chatbots to help you study? (e.g., clarifying content, coming up with points, explanations, writing content, etc.) Yes No Q7. How long have you used AI LLM Chatbots? 1–3 months 4–6 months 7–9 months 10–12 months Under 2 years Over 2 years Q8. How frequent do you use AI LLM Chatbots per week for studying? 1 time per week 2 times per week 3 times per week 4 times per week 5 times per week 6 times per week 7 times per week (every day) Never/Rarely Q9. Which AI-powered chatbots have you used? (Select all that apply) ChatGPT Google Gemini Microsoft Copilot / Bing AI Meta’s Llama Other… Q10. Which of the following best describes how you use LLM chatbots in your studies? (Select all that apply) Understanding complex concepts Generating study materials (e.g., summaries, flashcards) Assisting with problem-solving or assignments Reviewing or editing written work Writing essays/paragraphs Preparing for exams Other… Q11. How confident are you in your ability to use AI-powered chatbots effectively? Not confident at all Very Confident 1 2 3 4 5 Q12. Please indicate your level of agreement with the following statements Strongly Disagree Disagree Neutral Agree Strongly Agree (a) LLM chatbots are helpful in enhancing my learning experience. (b) LLM chatbots provide accurate information relevant to my studies. (c) Using LLM chatbots has improved my academic performance. (d) I find LLM chatbots easy to use. (e) I am concerned about the reliability of information provided by LLM chatbots. (f) LLM chatbots encourage me to think critically about the information I receive. (g) I prefer traditional study resources over LLM chatbots. (h) LLM chatbots save me time in completing academic tasks. (i) I am worried about potential academic integrity issues when using LLM chatbots. (j) LLM chatbots will play a significant role in the future of medical education. Q13. How do you perceive the quality of responses provided by LLM chatbots? Very Poor Very Good 1 2 3 4 5 Q14. What limitations do you perceive in using AI-powered chatbots? (Select all that apply) Inaccuracy of information Difficulty prompting Difficulty in understanding complex queries Traditional Resources, including Google Search, are more than enough Lack of creativity Lack of depth in responses User interface or formatting is confusing Unclear Wordings Other… Q15. To what extent do you believe LLM chatbots can replace traditional learning resources (e.g., textbooks, lectures)? Not at all Completely 1 2 3 4 5 Q16. After using AI LLM Chatbots, how confident do you feel in applying the information learned? Not confident at all Very confident 1 2 3 4 5 Q17. What potential benefits do you see in integrating LLM chatbots into medical education? (Select all that apply) Personalized learning experiences Immediate access to information Enhanced understanding of complex topics Improved study efficiency Development of critical thinking skills Assistance with problem-solving Support for collaborative learning Other… Q18. In your opinion, how important is it for medical students to be proficient in using AI tools? Not Important Extremely Important 1 2 3 4 5 Q19. How likely are you to continue using AI LLM Chatbots in your future studies? Not likely at all Very likely 1 2 3 4 5

## Appendix B. Task 1 Questions

Part 1: Single Best Answer (SBA) Questions Question 1 A 24-year-old male presents to the emergency department after a motorcycle accident. He is unable to abduct his shoulder and has significant weakness when trying to externally rotate the arm. Physical examination reveals an inability to initiate shoulder abduction and a loss of sensation over the lateral aspect of the shoulder. Which part of the brachial plexus is most likely injured? A. Suprascapular and axillary nerves B. Long thoracic nerve C. Medial cord D. Musculocutaneous nerve E. Radial nerve Question 2 A 35-year-old construction worker reports weakness in his hand after a fall from a ladder. On examination, he exhibits wrist drop and weakened extension of the fingers and elbow. Sensory loss is noted on the posterior aspect of the forearm and dorsum of the hand. Which part of the brachial plexus is likely affected? A. Lateral cord B. Medial cord C. Posterior cord D. Suprascapular nerve E. Ulnar nerve Question 3 A 19-year-old college athlete presents with numbness and tingling along the medial side of the forearm and hand following an incident while lifting weights. Examination reveals weakness in flexing the fourth and fifth digits and diminished hand grip strength. Which nerve of the brachial plexus is most likely compressed or damaged? A. Axillary nerve B. Long thoracic nerve C. Median nerve D. Musculocutaneous nerve E. Ulnar nerve Question 4 A 32-year-old woman visits the clinic complaining of difficulty gripping objects and numbness along the lateral aspect of her palm and first three fingers. Upon examination, you notice she cannot make a fist, as the index and middle fingers remain extended while attempting to close her hand. What condition is she most likely suffering from? A. Axillary nerve damage B. Carpal tunnel syndrome C. Hand of benediction D. Radial nerve palsy E. Thoracic outlet syndrome Question 5 A 40-year-old office worker presents with tingling and numbness in his thumb, index, and middle fingers, especially at night. He also reports weakness when trying to perform pinching motions, such as holding a pen or turning a key. Physical examination shows atrophy of the thenar eminence. Which of the following is the most likely diagnosis? A. Carpal tunnel syndrome B. Cubital tunnel syndrome C. Hand of benediction D. Klumpke’s palsy E. Thoracic outlet syndrome Question 6 Which of the following signs is most consistent with cubital tunnel syndrome? A. Atrophy of the hypothenar and interosseous muscles B. Inability to extend the wrist, resulting in a wrist drop C. Loss of sensation over the lateral three and a half digits D. Weakness in shoulder abduction and external rotation E. Weakness in elbow flexion and forearm supination Question 7 A 25-year-old athlete presents with weakness when abducting the shoulder and externally rotating the arm. On examination, there is a notable flattening of the shoulder contour, and sensation is diminished over the lateral aspect of the upper arm. Which of the following clinical conditions is most likely? A. Erb-Duchenne palsy B. Klumpke’s palsy C. Radial nerve palsy D. Rotator cuff tear E. Shoulder dislocation-associated nerve injury Part 2: Short Answer Questions Question 1: What are the five terminal branches of the brachial plexus? Question 2: Which nerve of the brachial plexus is responsible for innervating the biceps brachii muscle? Question 3: A 34-year-old cyclist falls off his bike and lands on his outstretched arm. He presents with weakness in shoulder abduction, elbow flexion, and loss of sensation over the lateral aspect of the forearm. Which part of the brachial plexus is most likely affected? Question 4: A 50-year-old patient complains of difficulty with wrist and finger extension, resulting in a characteristic wrist drop. On examination, there is also numbness over the posterior aspect of the forearm and dorsum of the hand. Which nerve of the brachial plexus is most likely involved? Question 5: A 27-year-old weightlifter experiences pain and weakness when trying to lift his arm above his head. On examination, you notice scapular winging when he pushes against a wall. What condition related to the brachial plexus is most likely causing these symptoms? Question 6: A newborn is delivered via difficult labor and presents with a “claw hand” deformity affecting the wrist and fingers. The infant also exhibits weakness in the intrinsic muscles of the hand. Which condition related to the brachial plexus is most likely the diagnosis? Question 7: A 45-year-old man presents with a history of numbness and tingling in the fourth and fifth fingers, along with weakness in finger abduction and adduction. He also has atrophy of the hypothenar muscles. What condition related to the brachial plexus could explain these findings?

## Appendix C. Task 2 Questions

Part 1: Single Best Answer (SBA) Questions Question 1: Which nerve, originating from the lumbar plexus, is responsible for innervating the quadriceps muscle group? A. Femoral nerve B. Obturator nerve C. Pudendal nerve D. Sciatic nerve E. Superior gluteal nerve Question 2 (this question is cancelled) A 50-year-old woman presents with difficulty climbing stairs and standing up from a seated position. She also reports weakness in hip extension. Which nerve is most likely affected? A. Femoral nerve B. Inferior gluteal nerve C. Obturator nerve D. Sciatic nerve E. Superior gluteal nerve Correct answer: B. Inferior gluteal nerve Question 3: A 45-year-old man reports pain radiating from his lower back to the posterior aspect of his thigh and down to his calf and foot. He also has difficulty with knee flexion and dorsiflexion of the foot. Which nerve is most likely implicated in this case? A. Femoral nerve B. Inferior gluteal nerve C. Obturator nerve D. Sciatic nerve E. Superior gluteal nerve Question 4 A 36-year-old woman presents to the clinic with a complaint of difficulty walking, particularly when stepping out of a car. On examination, she has weakness in thigh adduction and a sensory deficit over the medial aspect of her thigh. She denies any back pain. What nerve is most likely impaired? (A) Common peroneal nerve (B) Femoral nerve (C) Inferior gluteal nerve (D) Obturator nerve (E) Tibial nerve Question 5 A 45-year-old woman presents to the emergency department with acute onset of severe pain in the left lower limb, which started while she was climbing stairs. On examination, she has a palpable, tender mass in the popliteal fossa, and the pain increases with passive dorsiflexion of the foot. The patient has a history of chronic venous insufficiency. Which of the following conditions is the most likely cause of her symptoms? A. Achilles tendon rupture B. Deep vein thrombosis C. Femoral hernia D. Popliteal artery aneurysm E. Ruptured Baker’s cyst Question 6 Which of the following signs or symptoms is most characteristic of Charcot-Marie-Tooth disease? A. High-stepping gait with foot drop B. Claudication pain relieved by rest C. Glove and stocking sensory loss D. Severe pain and swelling after trauma E. Sudden, sharp back pain radiating down the leg Question 7 A 72-year-old man presents with pain in the left hip that radiates down the lateral aspect of the thigh to the knee. He has difficulty walking and frequently loses his balance. On examination, there is weakness in hip abduction, and Trendelenburg’s sign is positive on the left side. Which of the following is the most likely cause of his symptoms? A. Greater trochanteric pain syndrome B. Iliotibial band syndrome C. Lumbar spinal stenosis D. Meralgia paresthetica E. Osteoarthritis of the hip Part 2: Short Answer Questions Question 1: Which nerve originating from the lumbosacral plexus is responsible for motor innervation of the quadriceps muscle group? Question 2: A 30-year-old man presents with weakness in hip flexion and knee extension after a motor vehicle accident. He also complains of numbness over the anterior thigh. Which nerve of the lumbosacral plexus is likely injured? Question 3: A 50-year-old woman complains of pain radiating from her lower back to the posterior thigh and lateral aspect of the leg. On examination, she has weakness in plantarflexion and absent ankle reflex. Which nerve of the lumbosacral plexus is affected? Question 4: A 25-year-old male athlete presents with difficulty standing on his toes and sensory loss over the sole of his foot. He denies any lower back pain. What is the most likely nerve involved, and what could be a potential cause? Question 5: A 65-year-old woman with a history of hip replacement surgery reports weakness in hip abduction and numbness over the lateral aspect of her thigh. Which nerve of the lumbosacral plexus may have been damaged during the surgery, and what symptoms support this diagnosis? Question 6: A 45-year-old man presents with severe pain in his lower back that radiates down the posterior thigh and into the lateral aspect of his foot. On physical examination, he has difficulty with ankle dorsiflexion and reduced sensation over the dorsum of the foot. Which nerve root is most likely compressed, and what condition is commonly associated with this presentation? Question 7: A 60-year-old woman who recently underwent pelvic surgery presents with difficulty climbing stairs and weakness in extending her knee. She also complains of numbness over the anterior and medial thigh. Which nerve is most likely injured, and what is a potential complication related to this nerve damage?

## Appendix D. Post Task 1 Questionnaire

QVault AI Chatbot Study—Post task 1 questionnaire Dear Participant, Thank you for completing Task 1 of our study. Please take a few minutes to complete this questionnaire about your experience during the task. Your responses are crucial for our research on learning methods in medical education. This questionnaire should take approximately 5 min to complete. Your responses are confidential and will be used solely for research purposes. Q1. Assigned ID number Section 1: Instructions Please answer the following questions based on the learning method you used during Task 1: Group A: Used the LLM chatbot along with the provided handout. Group B: Used traditional resources (e.g., Google search without AI features and textbook handout). Section 2 of 5 Section A: Experience with the learning method Q2. How easy was it to used the learning method provided during Task 1? Very difficult Very easy 1 2 3 4 5 Q3. How satisfied are you with the learning method you used during Task 1? Very dissatisfied Very satisfied 1 2 3 4 5 Q4. To what extent did the learning method help you understand the topic (brachial plexus) during task 1? Not at all A great deal 1 2 3 4 5 Q5. How efficient was the learning method in helping you find the information you needed? Very Inefficient Very efficient 1 2 3 4 5 Q6. After using the assigned learning method, how confident do you feel in applying the information learned? Not confident at all Very confident 1 2 3 4 5 Section 3 of 5 Section B: Perceptions of the learning method Q7. How would you rate the quality of the information provided by your assigned learning method? Very poor Excellent 1 2 3 4 5 Q8. To what extent do you believe the information obtained from your given learning method was accurate? Not accurate at all Completely Accurate 1 2 3 4 5 Q9. How would you rate the depth of content provided by your given learning method? Very superficial Very in-depth 1 2 3 4 5 Q10. How easy was it to understand the information provided by your given learning method? Very difficult Very easy 1 2 3 4 5 Q11. How engaging was the learning method in maintaining your interest during the task? Not engaging at all Extremely engaging 1 2 3 4 5 Section 4 of 5 Section C: Comparison with previous learning experiences Q12. Compared to your usual study methods, how did the learning method you used during Task 1 perform? Much worse Much better 1 2 3 4 5 Q13. How do you feel the learning method affected your ability to think critically about the subject matter? Strongly hindered Strongly aided 1 2 3 4 5 Q14. How likely are you to use this type of learning method in your future studies? Very Unlikely Very likely 1 2 3 4 5 Section 5 of 5 Section D: Perceptions of LLM chatbots Q14. What is your overall attitude toward the use of LLM chatbots in medical education? Very negative Very positive 1 2 3 4 5 Q15. How useful do you believe LLM chatbots are in supporting medical education? Not useful at all Extremely useful 1 2 3 4 5 Q16. Do you have any concerns about using LLM chatbots in your studies (Select all that apply) Accuracy of information Over reliance on technology Ethical considerations Privacy issues Impact on critical thinking skills Lack of human interaction No concerns Other… Q17. How interested are you in using LLM chatbots in your future studies? Not interested at all Extremely Interested 1 2 3 4 5

## Appendix E. Post Task 2 Questionnaire

QVault AI Chatbot Study—Post task 2 questionnaire Dear Participant, Thank you for completing Task 2 of our study. Please take a few minutes to complete this questionnaire about your experience during the task. Your responses are crucial for our research on learning methods in medical education. This questionnaire should take approximately 5 min to complete. Your responses are confidential and will be used solely for research purposes. Assigned ID number Section 1: Instructions Please answer the following questions based on the learning method you used during Task 2: Group A: Used traditional resources (e.g., Google search without AI features and textbook handout). Group B: Used the LLM chatbot along with the provided handout. Section 2 of 6 Section A: Experience with the learning method Q1. How easy was it to use the learning method provided during Task 2? Very difficult Very easy 1 2 3 4 5 Q2. How satisfied are you with the learning method you used during Task 2? Very dissatisfied Very satisfied 1 2 3 4 5 Q3. To what extent did the learning method help you understand the topic (lumbosacral plexus) during task 2? Not at all A great deal 1 2 3 4 5 Q4. How efficient was the learning method in helping you find the information you needed? Very Inefficient Very efficient 1 2 3 4 5 Q5. After using the assigned learning method, how confident do you feel in your understanding of the lumbosacral plexus? Not confident at all Very confident 1 2 3 4 5 Section 3 of 6 Section B: Perceptions of the learning method Q6. How would you rate the quality of the information provided by your assigned learning method? Very poor Excellent 1 2 3 4 5 Q7. To what extent do you believe the information obtained from your given learning method was accurate? Not accurate at all Completely Accurate 1 2 3 4 5 Q8. How would you rate the depth of content provided by your given learning method? Very superficial Very in-depth 1 2 3 4 5 Q9. How easy was it to understand the information provided by your given learning method? Very difficult Very easy 1 2 3 4 5 Q10. How engaging was the learning method in maintaining your interest during the task? Not engaging at all Extremely engaging 1 2 3 4 5 Section 4 of 6 Section C: Comparison with previous learning experiences Q11. Compared to your usual study methods, how did the learning method you used during Task 2 perform? Much worse Much better 1 2 3 4 5 Q12. How do you feel the learning method affected your ability to think critically about the subject matter? Strongly hindered Strongly aided 1 2 3 4 5 Q13. How likely are you to use this type of learning method in your future studies? Very Unlikely Very likely 1 2 3 4 5 Section 5 of 6 Section D: Overall Perceptions After Using Both Learning Methods Q14. After using both learning methods (LLM chatbot and Traditional resources), which do you prefer? Strongly prefer traditional resources Strongly prefer LLM chatbot 1 2 3 4 5 Q15. Which learning method do you feel improved your understanding of the topics more effectively? Traditional Resources LLM chatbot Both equally Neither Q16. Do you anticipate changing your study habits based on your experiences with these learning methods? Not at all Extremely 1 2 3 4 5 Q17. How likely are you to recommend the use of LLM chatbots to your peers for studying medical topics? Very unlikely Very likely 1 2 3 4 5 Section 6 of 6 Section E: Perceptions of LLM chatbots Q18. What is your overall attitude towards the use of LLM chatbots in medical education after completing both tasks? Very negative Very positive 1 2 3 4 5 Q19. How useful do you believe LLM chatbots are in supporting medical education after your experiences in this study? Not useful at all Extremely useful 1 2 3 4 5 Q20. How interested are you in using LLM chatbots in your future studies after participating in this study? Not interested at all Extremely interested 1 2 3 4 5 Q21. Do you have any concerns about using LLM chatbots in your studies after your experiences in this study? (Select all that apply) Accuracy of information Over-reliance on technology Ethical considerations Privacy issues Impact on critical thinking skills Lack of human interaction No concerns Other…

## Appendix F. Focus Group Transcripts

Day 1 Focus Group 1 (20 November) 1. Describe your experiences using chatbot and traditional experiences Took a lot longer to filter through information in traditional. Chatbot was faster Traditional gave you everything Enjoyed a mix of both Speed—depended on the type of question—conditions you didn’t know—then traditional would have been quicker. But if it was single answer then chatbot was The aim was to get the answer down, not to learn the information, he won’t remember the information at all. Main goal was Chatbot was useful because it could provide and double check the answer, to verify it was helpful. When they didn’t have the chatbot he had to use the traditional sources and notes, Chatbot took some time to respond, some of the time was spent waiting for the chatbot to respond. Critical thinking questions—googling and using notes enhanced the critical thinking instead of in the chatbot. Chatbot didn’t encourage too much critical thinking. Very med student friendly as it gave mnemonics. Were there any surprises? - Character limit - The mnemonic function—was a good thing but it would be even better if it had a toggle to switch off the mnemonic function (as not everything needs mnemonics)—Sometimes the reply didn’t really work - Cannot send another question while the chatbot is thinking 2. How did your thoughts evolve about using (3) Overall quite positive, not generalised like chat gpt and it was customisable which was good, sometimes gives too much information which I don’t necessarily need for the lecture. Don’t know if the information is legit using general chatGPT, but this one seems more legit. (2) Was surprised to be able to use a chatbot for reputable information within the first few years of university, instead of using textbooks. (4) You usually have to spend a lot of time refinding your prompts for chatGPT, but Lenny gives you the relevant med school related information as the prompt is already refined. 3. Question 3 (1) Traditional—you have to go through all of the information, and the residual information is quite useful for understanding, whereas with a chatbot you can ask the specific question. Chatbot is better for specific questions but traditional (2) Not sure how you would use the chatbot to understand a whole topic, feels like chatbot is good for filling in the gaps in the lecture knowledge. 4. How did each learning method impact understanding and retention (2) The nature of the exercise made it so that he would just get the answer, and not enough time to actually understand the information. This method didn’t allow retaining the information. (3) The nature of the tasks today—you have time constraints so it was a bit hard to concentrate on learning. And also anatomy is more about memorising, not understanding. (4) If the purpose was learning over answering questions, then we would have learned a bit more. We didn’t know if the answers we got were correct at all, and because of the time con Did you try to get the right answer or were you just trying to fill in the boxes? Both, mostly because of the time constraint but also wanted to get the questions right. 5. How would you evaluate… usability etc. (4) Naturally engagement with traditional methods would allow more learning. There is merit in the process of finding out the information for yourself and learning things in the process, as you are being curious about the topic and engaging with the resources more. However Lenny is good at (1) Also need to find reputable sources through traditional sources, otherwise you’re not going to get the right answer. By sifting through the materials by yourself and diving into other topics at the same time you learn more. (3) Lenny is more question and answer, question and answer, so there may not be as much understanding. 6. Any specific features? (3) Mnemonics—see that could be good in memorising, other features of qVault could be very useful as well, e.g., podcasts, anki generation etc. 7. Challenges or limitations? (4) lack of critical thinking with the chatbot—a little bit of surrounding information would be good. If she got another random question on the same topic, she wouldn’t know how to answer the questions. (Isaac says this is more of a resource constraint but acknowledges this point). But a good thing is that you don’t have to refine the prompt in order to get a medical school level answer. (2) could be useful to show diagrams—the chatbot didn’t have diagrams. He feels like the delay is okay for a while, but if he were to use it for a long time it could be frustrating to use. One feature to improve on is the scrolling aspect—it should scroll to the bottom (didn’t happen for two people) (3) Interface—the follow up between the questions is good in chatGPT, but maybe not in Lenny. Even when you used the reply function, the responses were not specific to the follow up question. Also there is a delay. Would prefer instantaneous responses and 8. How did your experience with it influence your view? (4) More inclined to use Lenny if it is more refined in the future, if it deals with the delays and also delves into a little bit more surrounding it. But is happy about the specificity of Lenny and how it can refine to medical school standard (2) in its current state would only use it from time to time, but will look out for similar models which would be useful for exams. Would be useful to go through the missing parts of exams just to fill in the knowledge missing from lecture slides or clarify things that they are unsure about. (1) Previously would not have been inclined to use ChatGPT or AI related sources 9. Suggestions for improvement - Anki function - Fix delay if possible - Mnemonic toggle - Diagrams or pictures - Maybe reputable sources for the trust aspect - Question generation 10. What roles do you envision—how could they enhance existing learning methods? (2) The ideal scenario is to upload the powerpoint or transcript, and it could generate ankis and questions, and include diagrams—so you can do spaced repetition and also test your knowledge. Also test your own knowledge over time and see progress. (4) Being able to generate a whole booklet of questions would be really useful. (3) good quality questions—maybe collaborate with the universities/med schools to make sure the content is more accurate. 11. Anything you want to add? (4) The UI is very clean, easy to use, and despite the delays it was very good to use. Bearable for the purposes of this task. (1) King’s specific marketing is also good (2 and 3)—I like Lenny. Day 1 Focus Group 2 (20 November) Key Themes and Insights 1. Experiences Using Chatbot and Traditional Resources Chatbot give concise responses vs. Google searching gives complicated info (3) Hard to search directly for the answer in Google, but in chatbot it gives a direct answer (3) AI simplifies process to gain info, but need to learn how to use AI too, or else not helpful in knowledge retention (1) Traditional: all material given, can see what is related Chatbot needs user input and cannot see how everything is related, more useful when specific query in mind instead of learning entire topic Chatbots particularly useful in diagnosis questions vs. google Need to keep chatbot content accurate (4) Best to train it to be tailored to curriculum—ensure relevance (4) More useful if add reference in chatbot responses—credibility (everyone agrees) 2. Speed and Retention Latency—discourages users, may just sway people back to Google search (1,4) Waiting time/notifications/technical issues may disrupt learning 3. AI Handouts Handouts have distinct details, much quicker to get details than chatbot (only direct factual questions, chatbot better in diagnosis questions) (4) Liked how the handout give only relevant info (4) AI-generated handout vs. textbook: Handouts for basic knowledge, use textbooks for extra info (1) 4. Evolution of Thoughts on Chatbots Little chatbot exposure before, only the AI overview in Chrome, more open to using chatbot after this (2) Will only use chatbots when confused with medical concepts instead of learning new concepts (3) 5. Learning and Retention Understand lecture slides: use anki (4) AI for understanding harder topics (4) Only use chatbot when confused with something (2) More useful only in consolidating already learnt basic knowledge (3) Useful in learning & understanding concepts, not necessarily lead to better exam results (4) Chatbots may make students lazy in learning, requires discipline to memorize content (2,3) 6. Specific Features and Challenges Mnemonics (only) sometimes useful (1,3) interested in the AI question generation feature—Better than passmed since they are personalised to uploaded lectures Add diagrams, Anki generation, and question creation. 7. Roles in Learning Methods Still trust question banks more than AI-generated questions (1) Will trust AI more if it is trained based on past papers (all agree)—since users do not have knowledge whether all the content is true 8. Others Best to make it free to be accessible to everyone (1) (discussed cost issues) Day 2 Focus Group 3 (11 Dec) 1. Experiences with Learning Methods Can you describe your experiences using both the LLM chatbot and traditional resources during the tasks? Probe: What stood out to you about each method? Were there any surprises? 001: - More engaging than safari or google and more personalised - AI responds to exactly what you ask, but google will give a general response to similarly asked questions 003: - So much info in google/ safari, so in AI, it is more concise and everything is in one thing - Google was giving loads of information, some also contradictory -> e.g., giving 2 separate answers - Makes it easier to learn a list of conditions - Less helpful to learn one specific thing/concept in detail, but to learn e.g., many symptoms is better (memorisation easier to learn using AI than understanding) 004: - Do not like mnemonic, as it enforces brute memorisation rather than understanding 005: - AI gives mnemonics to help learn and remember, and finds it useful 2. Perception Changes How did your perceptions of LLM chatbots evolve, if at all, from before the study to after using them during the tasks? Probe: What specific experiences influenced your opinions positively or negatively? 001: - Agree with 005, same points 003: - Started using GPT whilst using passmed, to make the passmed explanations simpler and shorter - Worried about inaccuracies, but if AI is specific enough, then answers seem to be more accurate 004: - AI more tailored than google 005: - Trust qvault more than chatgpt as it is tailored towards medical knowledge as chatgpt is more general 3. Comparative Effectiveness In what ways did the LLM chatbot and traditional resources differ in helping you understand medical topics? Probe: Can you give examples of when one method worked better than the other? 001: 003: - Google is frustrating when it gives contradictory answers, so she uses AI to confirm the case 004: - Nice introduction to a topic as you can read a concise description - Can double check with using AI, as you can ask “are you sure this is correct?” 005: - Chatbot for learning first time easier as it can give an overview before being swarmed with information 4. Impact on Learning How did each learning method impact your understanding and retention of the material? Probe: Did one method encourage deeper learning or critical thinking? Why? (Moderator’s Note: Probe carefully to distinguish between perceived and actual retention.) 001: - QV is a bit longer than GPT, but it is similar and not long enough to discourage from using QV 003: - Used mostly passmed, but also some youtube - Gpt can also exclude additional details not needed - Takes longer than GPT to generate but answers are better and more relevant 004: - QV Slower than GPT - Don’t like mnemonics 005: - Use osmosis, lectures and anki - QV slower than GPT - Would be better if there was an upload feature on QV 5. Usability and Engagement How would you evaluate the usability and engagement levels of the LLM chatbot compared to traditional resources? Probe: Were there any specific features that enhanced or detracted from your learning experience? (Moderator’s Note: Encourage discussion of both usability and engagement to balance the responses.) 001: - QV gives more information so makes life easier 003: - QV is really good, but the problem is even with rephrasing questions, she gets the same response so it is annoying 004: - Finds more useful than google - More concise than google 005: - Also finds more useful than google 6. Challenges Encountered What challenges or limitations did you face when using the LLM chatbot and traditional resources? Probe: How did you navigate or overcome these challenges? Did either method create unique difficulties? 001: - Prompt wrong, so takes more time as he needs to generate a new question so cannot finish - Agree with bullet point 3 of 003 003: - Cannot finish for both, as QV takes too long to generate - Keep questions as short as possible to generate questions faster - If unlimited time, can retain more information as information is the same as it is all tailored towards medicine so answers vary less if irrelvant 004: - Learnt how to use QV better so could improve with timing 005: - Not enough time to finish both, but feels with GPT she would have finished as it is faster - Word limit so could not ask whole question, so had to segment up one question into smaller bits - Feels that with time pressure, she learnt not much, as it was just regurgitation (no understanding or memory gained) 7. Influence on Future Study Habits How might your experiences with LLM chatbots influence your future study habits or strategies? Probe: Do you see yourself integrating AI tools into your routine, and if so, how? 001: - Will use more in future 003: - Very keen to use QV more in future and “would recommend to a friend” - Would use to learn content and also revise 004: - Will use more for revision 005: - Will use more for revision 8. Role of AI in Medical Education What potential roles do you envision for LLM chatbots in medical education? Probe: How could they complement or enhance existing learning methods? 001: - Does not know much about OSCE so did not comment much 003: - Thinks QV could help with OSCE with remembering guidelines or policies but less so with the communications skills or the human–human interaction 004: - Talking to real people is better than AI for OSCE practise as it is hard to simulate 005: - AI/QV could generate scenarios for OSCE practise - If it generates a mark scheme on the scenario, it could be used to practise the content 9. Suggestions for Improvement (Optional: Ask if time allows.) What improvements would you suggest for integrating LLM chatbots into medical education to better support learning? Probe: Are there specific features or functionalities you think would make them more effective? 001: - Faster will be better - Add image interpretation function 003: - Faster will be better - UI and layout is very good -> modern and sleek -> easy to use 004: - Faster will be better - Give citations and references will be better 005: - Faster will be better - Increase word count as it is a little bit restricting (e.g., 500 words) - Give citations and references will be better

## Appendix G. Focus Group Question Set

1. Experiences with Learning Methods - Can you describe your experiences using both the LLM chatbot and traditional resources during the tasks? Probe: What stood out to you about each method? Were there any surprises? 2. Perception Changes - How did your perceptions of LLM chatbots evolve, if at all, from before the study to after using them during the tasks? Probe: What specific experiences influenced your opinions positively or negatively? 3. Comparative Effectiveness - In what ways did the LLM chatbot and traditional resources differ in helping you understand medical topics? Probe: Can you give examples of when one method worked better than the other? 4. Impact on Learning - How did each learning method impact your understanding and retention of the material? Probe: Did one method encourage deeper learning or critical thinking? Why? (Moderator’s Note: Probe carefully to distinguish between perceived and actual retention.) 5. Usability and Engagement - How would you evaluate the usability and engagement levels of the LLM chatbot compared to traditional resources? Probe: Were there any specific features that enhanced or detracted from your learning experience? (Moderator’s Note: Encourage discussion of both usability and engagement to balance the responses.) 6. Challenges Encountered - What challenges or limitations did you face when using the LLM chatbot and traditional resources? Probe: How did you navigate or overcome these challenges? Did either method create unique difficulties? 7. Influence on Future Study Habits - How might your experiences with LLM chatbots influence your future study habits or strategies? Probe: Do you see yourself integrating AI tools into your routine, and if so, how? 8. Role of AI in Medical Education - What potential roles do you envision for LLM chatbots in medical education? Probe: How could they complement or enhance existing learning methods? 9. Suggestions for Improvement (Optional: Ask if time allows.) - What improvements would you suggest for integrating LLM chatbots into medical education to better support learning? Probe: Are there specific features or functionalities you think would make them more effective?
